# Progress in Surface and Interface Modification Strategies of MXene Materials for Energy Storage Applications

**DOI:** 10.3390/ma18153576

**Published:** 2025-07-30

**Authors:** Yizhao Han, Junhua Hu, Xinhong Liu, Fanfan Liu

**Affiliations:** 1 State Center for International Cooperation on Designer Low-Carbon & Environmental Materials (CDLCEM), School of Materials Science and Engineering, Zhengzhou University, Zhengzhou 450001, China; yizhao.han@outlook.com (Y.H.); hujh@zzu.edu.cn (J.H.); 2 Longmen Laboratory, Luoyang 471000, China

**Keywords:** interfaces, nanocomposite, batteries, intercalation engineering, surface functionalization, doping engineering, composite engineering

## Abstract

MXene materials have great potential for energy storage applications, owing to their unique two-dimensional structure, exceptional electrical conductivity, and versatile surface chemistry. However, the practical utilization of pristine MXenes is hindered by several intrinsic limitations, such as interlayer restacking (which impedes ion diffusion), susceptibility to oxidation in aqueous and oxygen-rich environments, instability of surface functional groups, and suboptimal electrical conductivity. The structural engineering and surface modification strategies of MXenes were reviewed in this manuscript. The modification approaches include intercalation, surface functionalization, doping, and composite engineering. The insights presented herein aim to promote the development and practical application of MXene-based materials in next-generation energy storage devices.

## 1. Introduction

MXenes, a family of two-dimensional transition metal carbides and nitrides, were first reported in 2011 by Gogotsi et al. through selective etching of the MAX phase Ti_3_AlC_2_ [1]. The resultant Ti_3_C_2_T_x_ displayed unique structural features, high electrical conductivity, and tunable surface chemistry, attracting extensive research interest in energy storage and conversion. Over the past decade, the MXene family has grown rapidly, offering diverse compositions and application potential. Despite their promising properties, pristine MXenes face several problems. These include layer restacking, which limits ion transport and surface area, poor environmental stability due to oxidation, and the presence of mixed surface terminations (–O, –OH, –F), which may impede conductivity and structural integrity [2]. These shortcomings significantly hinder their electrochemical performance, underscoring the need for targeted modification strategies [3,4,5].

**Figure 1:**
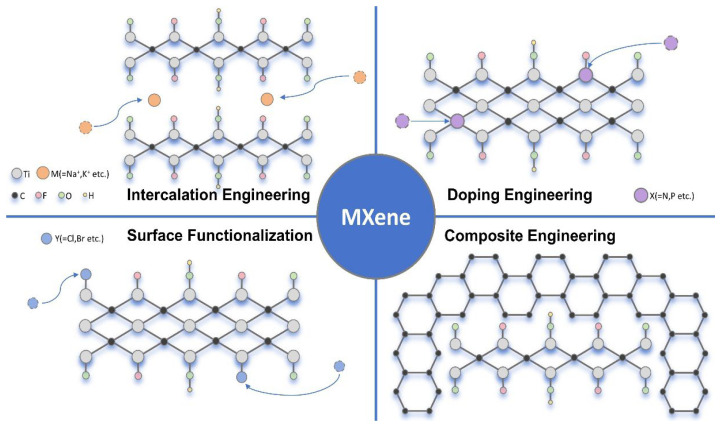
Schematic diagram of multi-engineering strategies for MXene structural optimization.

To solve the above problems, four primary modification strategies have been carried out (as shown in Figure 1): (1) intercalation engineering [6,7,8,9,10,11,12,13,14,15], (2) surface functionalization [16], (3) heteroatom doping [17,18], and (4) composite fabrication [19,20]. By inserting foreign species, such as metal ions (e.g., K^+^, Na^+^, Al^3+^), small organic molecules (e.g., dimethyl sulfoxide, urea), or even polymers, between MXene interlayers, this approach effectively enlarges the interlayer spacing, mitigates restacking, and facilitates rapid ion diffusion. The expanded interlayer channels not only enhance electrolyte accessibility but also improve charge storage kinetics, making intercalated MXenes particularly promising for high-rate battery applications. Strategic modification of the functional groups (e.g., replacing –F with –O or introducing nitrogen-containing functionalities) can fine-tune the electronic structure, enhance hydrophilicity, and optimize redox-active sites, thereby improving capacitance and cycling stability. Introducing foreign atoms (e.g., N, S, and P for non-metal doping; Ti, V, and Mo for metal doping) into the MXene lattice or surface can induce structural defects, modify charge distribution, and enhance intrinsic conductivity. Constructing hybrid architectures by integrating MXenes with conductive polymers, carbonaceous materials (graphene, carbon nanotubes), or metal oxides (TiO_2_, MnO_2_) synergistically combines their individual advantages [3].

Four modification strategies elucidate their underlying principles, structural influences, and consequent enhancements in electrochemical performance. This review presents the multi-scale integration of modification techniques, delivering both a state-of-the-art assessment and forward-looking design principles for the development of next-generation MXene-based energy storage systems.

## 2. Modification Strategies

### 2.1. Intercalation Engineering of MXene

Intercalation modification of MXene entails selecting appropriate ions, molecules, or polymers for insertion between the layers of the two-dimensional carbides or nitrides tailored to specific application requirements. These intercalants not only establish a supporting framework that prevents restacking and expands the interlayer spacing but also enhance ion-transport efficiency, thereby optimizing various electrochemical properties of MXene materials.

#### 2.1.1. Metal Ions and Their Oxides’ Intercalation Modification

**Figure 2:**
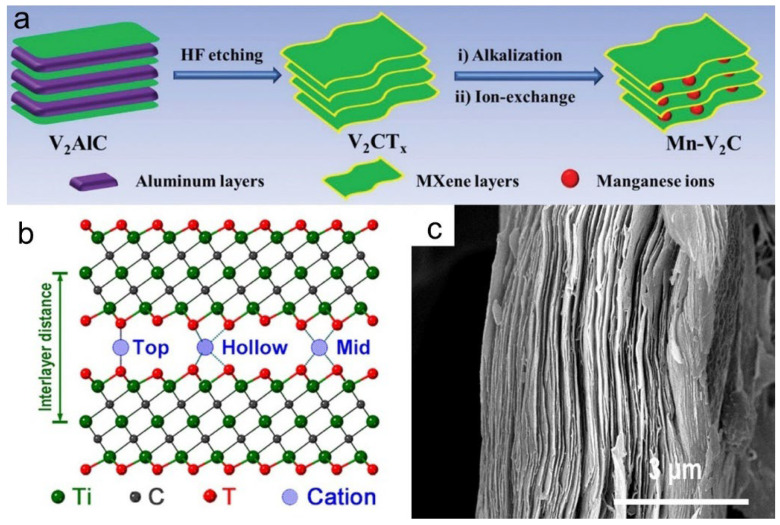
(**a**) Schematic illustration of the synthesis of Mn–V_2_C. Adapted from Ref. [10], with permission from Wiley-VCH. (**b**) Atomic scheme showing cation intercalated in Ti_3_C_2_T_x_ bilayer with three possible configurations. (**c**) Scanning electron microscopy (SEM) images of the cross-section of T-Mn-C. Adapted from Ref. [14], with permission from Elsevier.

V_2_CT_x_ MXene exhibits excellent electrical conductivity and a layered structure that facilitates ion transport. However, the low valence states of vanadium (V^2+^/V^3+^) in the original material and the small interlayer spacing limit its electrochemical activity. To address this, a cation intercalation strategy driven by electrochemical reactions was employed: V_2_CT_x_ was synthesized by HF etching of the V_2_AlC MAX phase, followed by KOH alkali treatment to induce ion exchange with Mn^2+^, forming Mn-V_2_C (Figure 2a). During the initial charge at 1.6 V, the outer layer of vanadium is oxidized to VO_x_, while the internal V_2_C conductive framework remains intact. This Mn^2+^ ion intercalation increases the interlayer spacing from 0.73 nm (in the original V_2_CT_x_) to 0.95 nm [10]. This method avoids the direct synthesis of highly toxic high-valence vanadium precursors by generating active species in situ via electrochemical processes. The resulting VO_x_/Mn-V_2_C material achieves a high capacity of 530 mAh·g^−1^ at 0.1 A·g^−1^, an energy density of 415 Wh·kg^−1^, and a power density of 5500 W·kg^−1^.

Moreover, it exhibits excellent cycling stability, retaining 84% of its capacity after 2000 cycles, with a Coulombic efficiency close to 100%. Mn^2+^ pre-intercalation effectively expands the interlayer spacing, stabilizes the structure, and enhances ion diffusion, confirming the benefits of metal ion intercalation. The optimal intercalation configuration varies with the cation type [14], as shown in the atomic model (Figure 2b). The cross-sectional SEM image of the T-Mn-C electrode (Figure 2c) reveals a well-maintained layered structure, indicating that cation intercalation and the subsequent calcination preserve the intrinsic 2D morphology of Ti_3_C_2_T_x_ MXenes.

**Figure 3:**
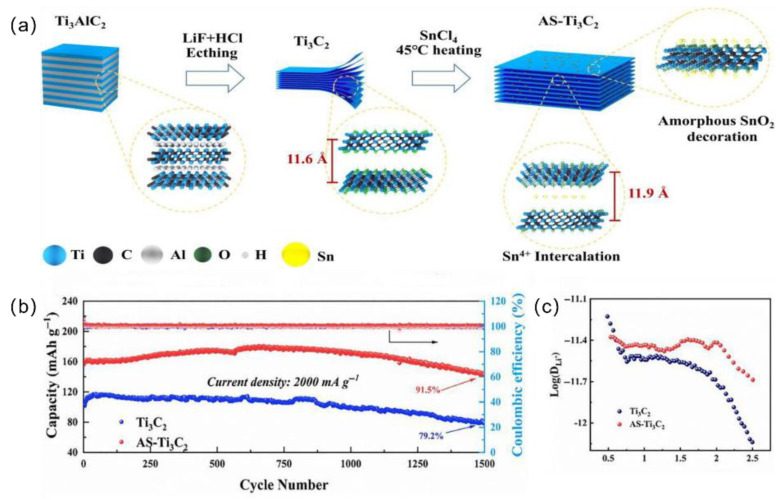
(**a**) Process of Sn^4+^ intercalation and amorphous SnO_2_ surface modification of AS-MXene materials. (**b**) Long-term cycling performance at 2000 mA·g^−1^. (**c**) Comparison of Li^+^ ion diffusion coefficients. Adapted from Ref. [13], with permission from Elsevier.

Sn^4+^, Na^+^, K^+^, Li^+^, and so on also exhibit effective intercalation properties [9,14,21]. For instance, by intercalating Sn^4+^ and modifying MXenes with SnO_2_, interlayer stacking is suppressed (Figure 3a), which enhances cycling stability (Figure 3b) and ion transport rate (Figure 3c), as well as increases ion storage capacity. The SnO_2_ layer serves as a structural support that increases interlayer spacing, reduces restacking, and improves the exposure of electrochemically active sites [9,13].

**Figure 4:**
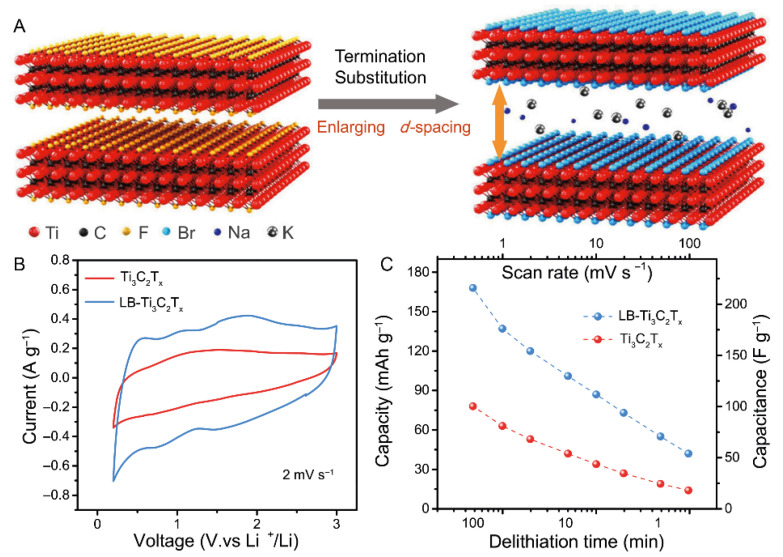
(**A**) Schematic illustration of the preparation of LB-Ti_3_C_2_T_x_. (**B**) Cyclic voltammetry curves of Ti_3_C_2_Tx and LB-Ti_3_C_2_T_x_ at a scan rate of 2 mV·s^−1^. (**C**) Comparison of the capacities of Ti_3_C_2_T_x_ and LB-Ti_3_C_2_T_x_ under various scan rates and delithiation times. Adapted from Ref. [22], with permission from Springer Nature.

Similarly, by treating Ti_3_C_2_T_x_ MXenes with NaOH, Na^+^ ions are intercalated, which increases the interlayer spacing. The specific capacitance of NaOH-treated Ti_3_C_2_T_x_ is significantly higher than that of untreated MXenes (specific capacitance increased from 61.3 F·g^−1^ to 113.4 F·g^−1^ at a scan rate of 1 mV·s^−1^), attributed to the enlargement of active sites by Na^+^ intercalation [7]. Additionally, by using a mixture of LiF and HCl to etch the Al layer in the V_2_AlC MAX phase, layered V_2_C MXenes are obtained. These acid-etched V_2_C MXenes are then immersed in a KOH solution, where K^+^ ions intercalate into the layers, further exfoliating the MXene nanosheets to form a porous structure (K-V_2_C). During the alkali treatment, K^+^ ions insert between the MXene layers via ion exchange or electrostatic interaction. K^+^ not only inserts alongside solvent molecules to form a stable intercalated complex, improving cycling stability, but also stabilizes the interlayer spacing by acting as a pillar, thus preventing re-stacking. The resulting porous structure promotes electrolyte penetration and ion diffusion [11,15]. Na^+^ and K^+^ ions were intercalated into Ti_3_C_2_T_x_ using a Lewis base halide treatment, effectively expanding the interlayer spacing and enhancing the electrochemical performance (Figure 4A).

In particular, the Langmuir–Blodgett (LB) technique, a classical method for assembling ultrathin films with highly ordered structures at the air–water interface, has been employed to fabricate LB-Ti_3_C_2_T_x_ films, offering precise control over the alignment and thickness of MXene layers. Furthermore, using an electrolyte containing Li^+^ (such as LiCl or Li_2_SO_4_), during charge and discharge, Li^+^ ions are driven by the electric field into the interlayers of the MXene structure. Li^+^ tends to occupy vacancies on the MXene surface formed by O/F atoms, and through electrostatic interaction, stabilizes the embedded Li^+^, increasing the interlayer spacing. The Li^+^ intercalation process effectively mitigates the problem of interlayer restacking, enhances ion transport kinetics (Figure 4B,C), and introduces additional pseudocapacitance, thereby improving the overall energy density and cycling stability of the material [8,12,14,22].

#### 2.1.2. Non-Metal Ion and Organic Molecule Intercalation Modification

**Figure 5:**
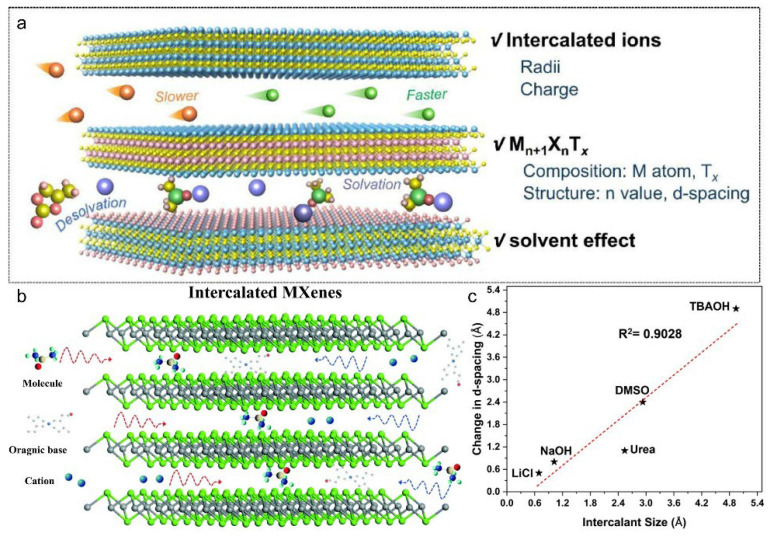
(**a**) Schematic illustration of influencing factors of ion intercalation. Adapted from Ref. [27], with permission from Elsevier. (**b**) Schematic illustration of non-metallic species intercalation into MXene layers. Adapted from Ref. [28], with permission from Royal Society of Chemistry. (**c**) Linear relationship between intercalant molecular size and interlayer spacing increase of Ti_3_C_2_T_x_. Adapted from Ref. [29], with permission from the American Chemical Society.

In addition to metal ions and their oxides, a variety of non-metal ions and organic molecules have been intercalated into MXenes to tailor their physicochemical properties. For instance, NH_4_^+^ intercalation expands the interlayer spacing of Ti_3_C_2_T_x_ from 19.8 Å to 24.5 Å, mitigating restacking and enhancing lithium-ion storage capacity to 168 mAh·g^−1^, compared to 100 mAh·g^−1^ for pristine Ti_3_C_2_T_x_ [23]. Similarly, ammoniation-derived Mo_2_Ti(C_1-δ_N_δ_)_2_T_x_(o-MXene) delivers an even higher capacity of 750 mAh·g^−1^ [24]. Post-etching intercalation using agents such as C_2_H_6_OS(DMSO), urea, and C_16_H_37_NO(TBAOH) facilitates delamination, suppresses restacking, and improves ion transport [25,26]. The polar sulfinyl group (S=O) of DMSO ((CH_3_)_2_S=O) forms hydrogen bonds with hydroxyl (-OH) or oxygen (-O) functional groups between MXene layers. TBAOH displaces interlayer H^+^ or small cations via ion exchange. These enhancements are governed by the intercalant’s size, charge, solvation state, and interactions with MXene surface terminations (Figure 5a).

For example, DMSO intercalation increases the c-lattice parameter from 19.5 Å to 26.8 Å, thereby promoting exfoliation [27]. As illustrated schematically, various types of non-metallic intercalants (e.g., neutral molecules, organic bases, and cations) can be accommodated between MXene layers, each inducing distinct degrees of spacing modulation and functional property tuning (Figure 5b) [28]. Urea and TBAOH effectively promote delamination, improving dispersibility and electrochemical performance. Notably, a quantitative correlation exists between intercalant size and interlayer spacing: Ti_3_C_2_T_x_ spacing increases from 12.0 Å to 15.0 Å with urea, and up to 19.0 Å with TBAOH, highlighting intercalant dimensions as a key design parameter in MXene structural engineering (Figure 5c) [29].

**Figure 6:**
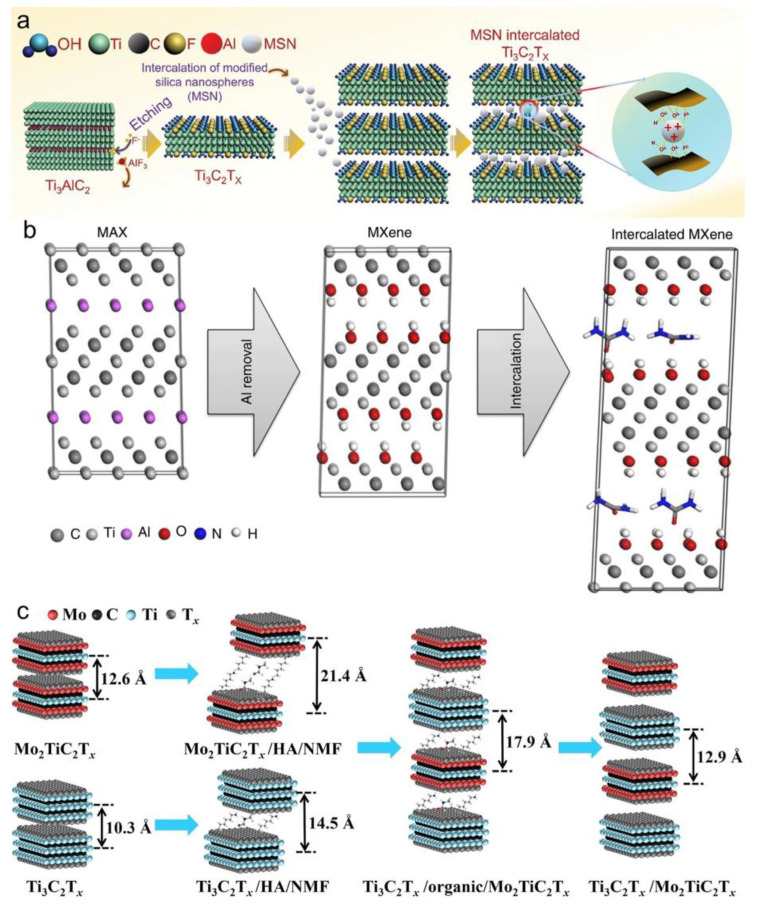
(**a**) Schematic illustration of MSN-intercalated Ti_3_C_2_T_x_ composite. Adapted from Ref. [6], with permission from Wiley-VCH. (**b**) Schematic illustration of the synthesis and intercalation modification of Ti_3_C_2_T_x_ MXene. Adapted from Ref. [30], with permission from Springer Nature. (**c**) Schematic illustration of the intercalation–deintercalation control strategy for Ti_3_C_2_T_x_/Mo_2_TiC_2_T_x_ heterostructure films. Adapted from Ref. [31], with permission from Elsevier.

In addition to conventional molecular intercalants, several advanced intercalation strategies have recently been developed. In 2024, Jinho et al. proposed a novel intercalation strategy to mitigate surface triboelectric charge dissipation and inhibit the self-restacking of MXene layers in triboelectric nanogenerators (TENGs). Specifically, modified silica nanospheres (MSNs) were intercalated between Ti_3_C_2_T_x_ layers, effectively increasing interlayer spacing and surface area, suppressing restacking, and enhancing both the dielectric constant and surface charge density. This modification significantly enhanced the output performance of TENGs, achieving a peak power density of 691.2 μW·cm^−2^, and also suggested a promising strategy for improving the charge storage in battery electrodes (Figure 6a) [6].

Organic molecules can be effectively intercalated into MXene interlayers through physical adsorption or chemical bonding, offering a versatile strategy for interlayer engineering. The abundant functional groups in small molecules and polymers enable various interactions, such as coordination or hydrogen bonding, with surface terminations, thereby stabilizing the interlayer architecture and expanding interlayer spacing. For example, as shown in Figure 6b, etching Ti_3_AlC_2_ with HF produces Ti_3_C_2_T_x_ MXene, which subsequently allows urea molecules to intercalate and interact via –NH_2_ groups, increasing the interlayer spacing from 19.5 Å to 25.48 Å and enhancing structural stability [30]. Beyond single-component systems, heterostructured intercalation strategies have been explored to fine-tune the interlayer distances and optimize the interface properties. For instance, alternating Ti_3_C_2_T_x_ and Mo_2_TiC_2_T_x_ layers enables precise modulation of the interlayer spacing from 12.6 Å to 21.4 Å, resulting in improved ion diffusion kinetics, volumetric capacitance, and structural integrity during cycling (Figure 6c) [31]. The intercalation of HA/NMF molecules enlarges the interlayer spacing of Ti_3_C_2_T_x_ and Mo_2_TiC_2_T_x_. Self-assembly yields a 17.9 Å intergap, reflecting organic-bridged synergistic effects. Direct stacking optimizes interfacial bonding, compressing spacing to 12.9 Å.

Beyond urea and heterostructured designs, a range of organic cations and small molecules have been employed to enhance MXene interlayer engineering. Although pristine Ti_3_C_2_T_x_ shows negligible Mg^2+^ storage due to poor intercalation, embedding cetyltrimethylammonium bromide (CTAB) expands the interlayer spacing, lowers the Mg^2+^ diffusion barrier, and improves ion transport [32]. Similarly, ethylenediamine (EDA) enables effective intercalation via N–Ti coordination, yielding a layered structure with a high capacitance of 356 F·g^−1^ and 92% retention after 10,000 cycles [9]. Alkylammonium (AA) cations with tunable chain lengths can also intercalate through cation exchange, forming stable AA–Ti_3_C_2_T_x_ complexes [33]. In addition, ionic liquids, such as 1-Butyl-3-methylimidazolium bis(trifluoromethanesulfonyl)imide(BuIMH-NTf_2_) and 1-Ethyl-3-methylimidazolium(EMIM-based) salts, can be incorporated via vacuum-assisted methods, offering further control over interlayer spacing and ion dynamics [34,35,36].

Collectively, these intercalation strategies highlight the significance of tailored interlayer engineering in optimizing both the electrochemical performance and long-term stability of MXene-based energy storage systems.

### 2.2. Surface Functionalization on MXene

MXenes typically possess surface terminations, such as –O, –OH, –F, and occasionally –Cl, which significantly influence their hydrophilicity, electronic structure, and interlayer interactions. However, these native terminations also introduce several limitations (such as poor environmental stability, reduced electrical conductivity, and restricted ion transport), which collectively hinder their functional performance. Therefore, the chemical and electrochemical modification of surface terminations has become imperative for optimizing the physicochemical properties of MXenes.

**Figure 7:**
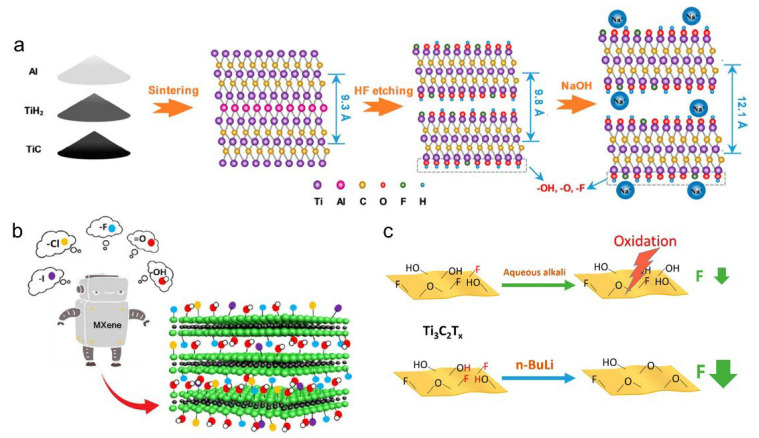
(**a**) Schematic illustration of surface functionalization of Ti_3_C_2_T_x_ MXene via HF etching and NaOH treatment. Adapted from Ref. [9], with permission from American Chemical Society. (**b**) Schematic representation of multisite functional group termination on MXene layers. Adapted from Ref. [40], with permission from Elsevier. (**c**) Schematic of the n-BuLi-modified and alkali-modified MXene synthesis. Adapted from Ref. [41], with permission from American Chemical Society.

Precisely tailoring the type and ratio of surface terminations is critical for optimizing the electrochemical performance of MXenes, as each functional group contributes unique physicochemical characteristics. The composition and abundance of these surface terminations are largely governed by the chemical potential of the etching agents (e.g., HF and LiF/HCl) and specific reaction parameters, such as temperature and duration. For instance, HF etching preferentially introduces –F groups, whereas the presence of water during the process facilitates the formation of –OH and =O terminations (Figure 7a) [9]. Additionally, the surface -OH groups present on natural deep eutectic solvents (NDES) can significantly enhance the hydrophilicity and ion transport efficiency of MXenes [37,38]. The –F terminations on MXenes are highly electronegative and tend to occupy active sites, thereby impeding ion transport and suppressing redox reactions. In contrast, –OH and =O terminal groups enhance the hydrophilicity of MXenes and promote more efficient ion transport [39]. Although –OH groups have long been considered intrinsic MXene terminations, Gao et al. suggested they may arise from environmental adsorption or by-products. Using a molten salt etching method (e.g., ZnCl_2_), they synthesized Cl-terminated MXenes as a safer alternative to HF etching and revealed that termination type and abundance significantly affect electronic conductivity (Figure 7b) [40]. For example, high-temperature annealing removes –F groups, improving conductivity, while increased =O content promotes alkali ion adsorption, enhancing capacity and kinetics in batteries such as lithium-ion systems.

The electrochemical performance of MXenes is strongly governed by the type and proportion of surface terminations, particularly –F, –OH, and =O groups. Sun et al. reported that high-temperature annealing combined with alkaline treatment effectively reduced the –F content while enriching oxygen-containing terminations, resulting in enhanced capacitive behavior [42]. Fan et al. further employed n-butyllithium (n-BuLi) to convert –F and –OH groups into –O groups, outperforming conventional alkaline treatments (e.g., KOH, NaOH, and LiOH) in preserving the structural integrity of Ti_3_C_2_T_x_. The introduction of –O terminations facilitated reversible H^+^ adsorption in acidic media, contributing to pseudocapacitance, whereas –F terminations were electrochemically inactive and impeded ion transport. Therefore, minimizing –F while enriching –O terminations effectively improves both the capacitance and cycling durability of MXene electrodes (Figure 7c) [41].

**Figure 8:**
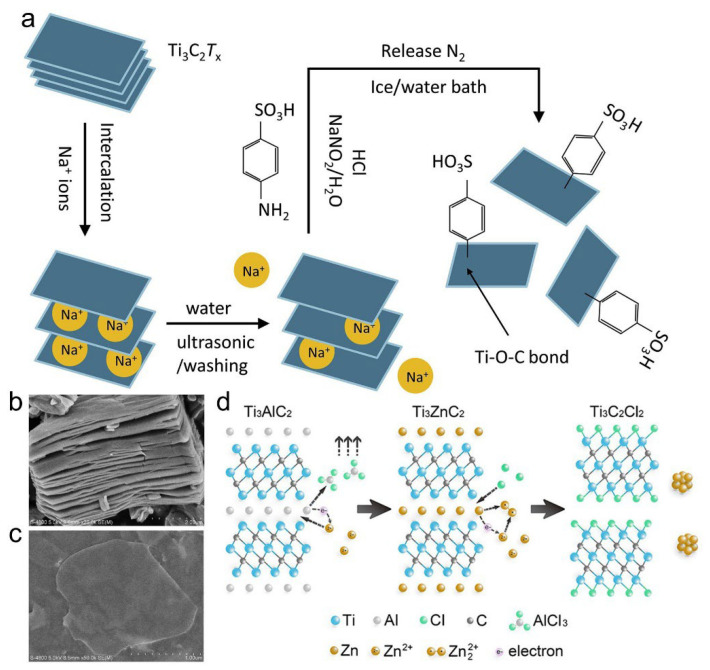
(**a**) Schematic representation of surface functionalization of Ti_3_C_2_T_x_ MXene via Na+ intercalation and diazonium-based aryl sulfonation. (**b**) Typical SEM image of the pristine Ti_3_C_2_. (**c**) SEM image of the functionalized Ti_3_C_2_. Adapted from Ref. [43], with permission from Elsevier. (**d**) Schematic illustration of the molten salt-mediated synthesis of Cl-terminated Ti_3_C_2_ MXene via Zn substitution and selective etching. Adapted from Ref. [44], with permission from American Chemical Society.

Building upon the intrinsic surface terminations of MXenes (–O, –OH, and –F), researchers have further investigated surface functionalization strategies, such as covalent grafting of organic moieties and halogenation, to finely tailor their physicochemical properties. For example, diazonium salts (e.g., sulfanilic acid diazonium salts) can generate aryl diazonium ions (Ar–N_2_^+^) in situ under acidic conditions, which subsequently form covalent bonds with oxygen-containing functional groups (e.g., –OH, –O) on the MXene surface. This reaction results in the formation of robust Ti–O–C linkages, thereby anchoring the organic groups onto the MXene framework (Figure 8a).

The SEM images demonstrate the morphological evolution of Ti_3_C_2_ MXene before and after surface functionalization, where the modified sample exhibits a more delaminated structure indicative of enhanced interlayer spacing and surface reactivity induced by functional group incorporation (Figure 8b,c). Functionalization with benzenesulfonic acid significantly improves dispersion and surface area, increasing from 18 m^2^·g^−1^ to 93.08 m^2^·g^−1^. As a result, the specific capacitance rises from 160 F·g^−1^ to 245 F·g^−1^ at 5 mV·s^−1^, while the capacitance retention reaches 92% after 10,000 cycles, highlighting improved electrochemical stability [43].

**Figure 9:**
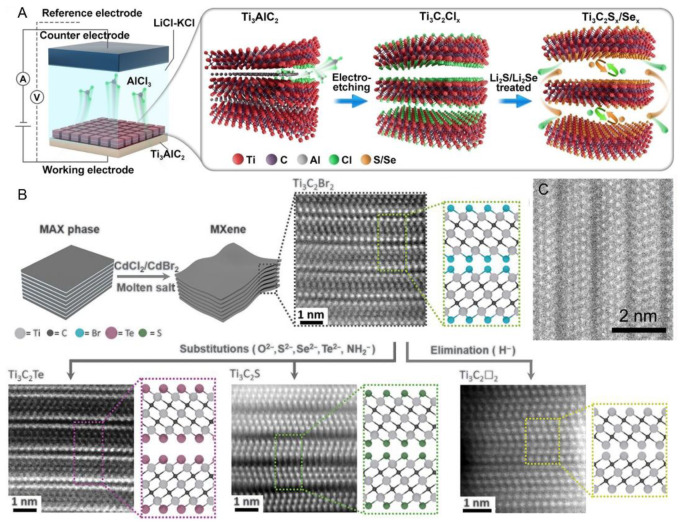
(**A**) Schematic diagram of the electrochemical etching and surface termination modification process of Ti_3_AlC_2_ to Ti_3_C_2_T_x_ (where T = Cl, S, or Se). (**B**) Schematic diagram and HAADF image of MXene structures with different surface termination groups (T = Br, Te, S, etc.) obtained by etching MAX phases in Lewis acidic molten salts. Adapted from Ref. [46], with permission from American Association for the Advancement of Science. (**C**) High-resolution atomic-scale high-angle annular dark-field (HAADF) image showing the layered structure of MXene. Adapted from Ref. [47], with permission from Wiley-VCH.

In addition to oxygen-containing functional groups, halogen terminations (e.g., –Cl, –Br) have also been widely employed to modify MXene surfaces. Huang et al. developed a universal approach to synthesize Zn-based MAX phases and Cl-terminated MXenes by leveraging a substitution reaction in molten ZnCl_2_, where Zn replaces Al in MAX precursors (e.g., Ti_3_AlC_2_, Ti_2_AlC, Ti_2_AlN, and V_2_AlC) (Figure 8d) [44]. This process yielded novel MAX structures (e.g., Ti_3_ZnC_2_, Ti_2_ZnC), which exfoliate under excess ZnCl_2_ due to its strong Lewis acidity, forming Cl-terminated MXenes such as Ti_3_C_2_Cl_2_ and Ti_2_CCl_2_ (Figure 9A). Subsequent substitution treatments with Li_2_S or Li_2_Se enable the formation of Ti_3_C_2_S_x_ and Ti_3_C_2_Se_x_, respectively [45]. Post-treatment of HF-etched MXenes with NaCl or NaBr also introduces Cl^−^ or Br^−^ terminations through covalent bonding, while hydrothermal and molten salt routes using copper halides (e.g., CuCl_2_, CuBr_2_) further expand the halogenation toolbox. Recently, Talapin et al. reported a molten salt-driven substitution–elimination platform capable of introducing or removing a wide range of terminations, including O, S, Se, Br, Te, and even imine, thus enabling fine-tuned control over MXene surface chemistry and structural integrity (Figure 9B,C) [46,47].

**Figure 10:**
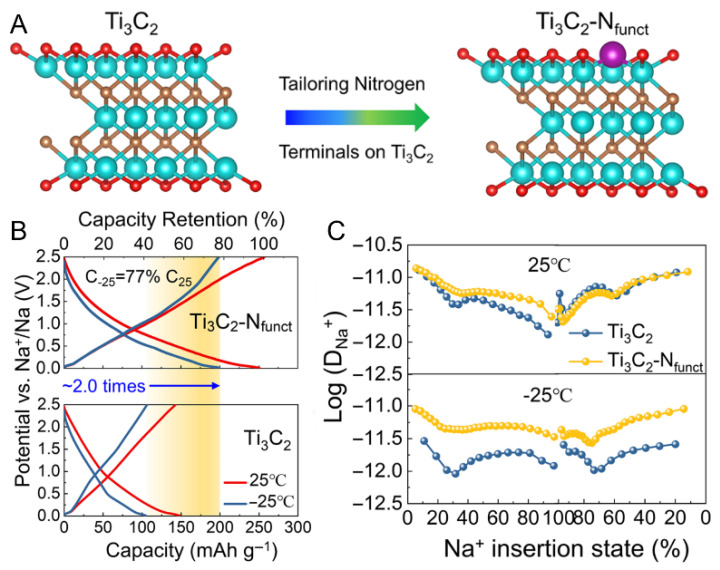
(Sodium-ion battery performance of Ti_3_C_2_ and Ti_3_C_2_-N_x_ anodes) (**A**) Schematic comparison of the structure and surface functional group design between Ti_3_C_2_ and Ti_3_C_2_-N_x_. (**B**) Second charge/discharge curves at 0.05 A·g^−1^ for Na-ion half-cells with Ti_3_C_2_ and Ti_3_C_2_-N_x_ anodes at 25 °C and −25 °C. (**C**) Comparison of Na^+^ diffusion coefficients for Ti_3_C_2_ and Ti_3_C_2_-N_x_ at 25 °C and −25 °C. Adapted from Ref. [48], with permission from Springer Nature.

To broaden the applicability of MXenes in diverse battery chemistries, researchers have proposed targeted surface functionalization strategies. For example, the introduction of nitrogen-containing functional groups into Ti_3_C_2_ significantly expands the interlayer spacing from 1.01 nm to 1.24 nm. This expansion facilitates improved ion diffusion and creates additional adsorption sites for sodium ions, collectively enhancing electrical conductivity and electrochemical performance (Figure 10A) [48].

The nitrogen-functionalized Ti_3_C_2_-N_x_ demonstrates markedly enhanced electrochemical performance in sodium-ion batteries, as evidenced by reduced Na^+^ diffusion barriers and lower charge transfer activation energies. Furthermore, Ti_3_C_2_-N_x_ exhibits a distinctive Na^+^ solvent co-intercalation mechanism at low temperatures, which effectively circumvents the high desolvation energy barrier. This behavior leads to higher energy density and outstanding cycling stability under both ambient and sub-zero temperature conditions (Figure 10B,C).

Furthermore, under vacuum conditions at 500 °C, zinc powder serves as a metallic reducing agent that reacts with surface –F terminations on MXenes via a displacement reaction. The residual zinc is subsequently removed through acid washing (e.g., with HCl), and –O functional groups are simultaneously introduced during the process [4]. Beyond thermal treatments, electrochemically driven redox reactions provide a powerful and reversible approach for atomic-level control over surface terminations. When a negative potential is applied, protons (H^+^) intercalate into the MXene interlayers and reduce =O terminations to –OH groups (e.g., Ti_3_C_2_O_2_ → Ti_3_C_2_(OH)_2_), thereby increasing the surface hydroxyl content. Conversely, a positive potential promotes deprotonation, partially oxidizing –OH back to =O and increasing the proportion of oxygen-based terminations. This reversible process can be described by the equation Ti_3_C_2_O_x_(OH)_y_^+^+δe^−^⇌Ti_3_C_2_O_x−δ_(OH)_y+δ_F_z_. This electrochemical approach optimizes the material properties for specific energy storage applications. While =O terminations enhance redox reactivity and are advantageous for high-capacity battery systems, –OH terminations facilitate ion diffusion and are, thus, better suited for high-power supercapacitors [49].

**Figure 11:**
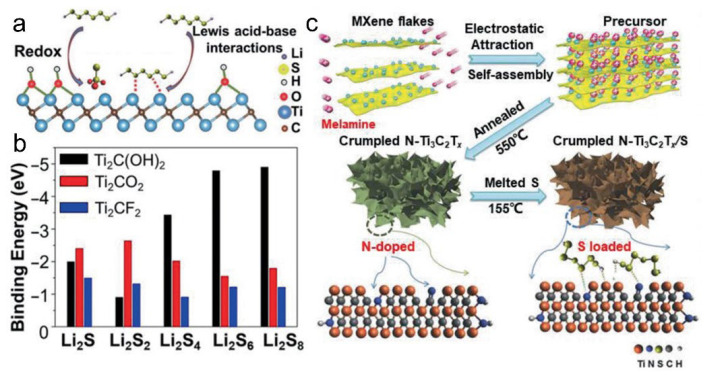
(**a**) Chemical entrapment of lithium polysulfides (LiPSs) by MXenes with –OH termination. (**b**) Binding energies of lithium polysulfides on the terminated Ti_3_C without electrolytes. (**c**) Schematic of the preparation of the crumpled N-doped Ti_3_C_2_T_x_ /S composite. Adapted from Ref. [52], with permission from Wiley-VCH.

In addition to electrochemical regulation, application-oriented surface functionalization has also been pursued to address specific challenges in emerging battery chemistries. For instance, MXenes have been employed as separator coatings to suppress polysulfide migration in sulfur-based batteries [50,51]. The presence of polar surface terminations such as –O and –F enhances the adsorption of polysulfides, effectively mitigating the shuttle effect and prolonging the cycling life of the battery. Beyond conventional surface terminations, the binding affinity and redox behavior of lithium polysulfides (LiPSs) are pivotal to the performance of lithium–sulfur batteries. Notably, –OH-terminated MXenes form strong Lewis acid–base interactions with LiPSs, effectively immobilizing polysulfides and mitigating the shuttle effect (Figure 11a,b).

Furthermore, melamine-assisted nitrogen doping followed by sulfur loading has been utilized to construct crumpled N-doped Ti_3_C_2_T_x_/S composites. This strategy not only introduces nitrogen-containing functional groups but also improves the structural stability and conductivity of the composite. The resulting material exhibits enhanced electrochemical performance due to the synergistic effects of nitrogen doping and sulfur anchoring, further underscoring the importance of surface termination engineering in MXene-based electrodes (Figure 11c) [52].

As previously discussed, the regulation of surface functional groups on MXenes typically begins with the selection of gas-phase or liquid-phase etchants (e.g., HF, LiF/HCl, and molten salts), which determine the introduction of specific terminations, such as –F, –OH, and –Cl, onto the MXene surface [45,52,53,54,55,56]. Post-synthetic modifications, including thermal annealing, heteroatom doping, and single-atom implantation, can further refine the surface termination chemistry and modulate the electronic structure of MXenes [52,57,58]. Alternatively, redox-driven or displacement reactions allow for the direct substitution of surface terminations, thereby enhancing interfacial adsorption capabilities and catalytic performance [46,47,49]. Collectively, these strategies provide versatile approaches to MXene surface termination engineering, facilitating the rational design of high-performance supercapacitors and a diverse array of metal-ion battery electrodes.

### 2.3. Doping Engineering on MXene

While intercalation and surface-functional group engineering improve MXene’s interlayer chemistry, they largely leave the bulk electronic framework unaltered—intercalants modulate inter-flake spacing but do not overcome intrinsic quantum-confinement effects or restore intra-flake conductivity. In contrast, heteroatom doping directly modifies the MXene lattice to create defect-rich ion channels, boost carrier density, and tune the Fermi level, yielding synergistic enhancements in electrical conductivity, ion transport kinetics, and overall electrochemical performance [3].

**Figure 12:**
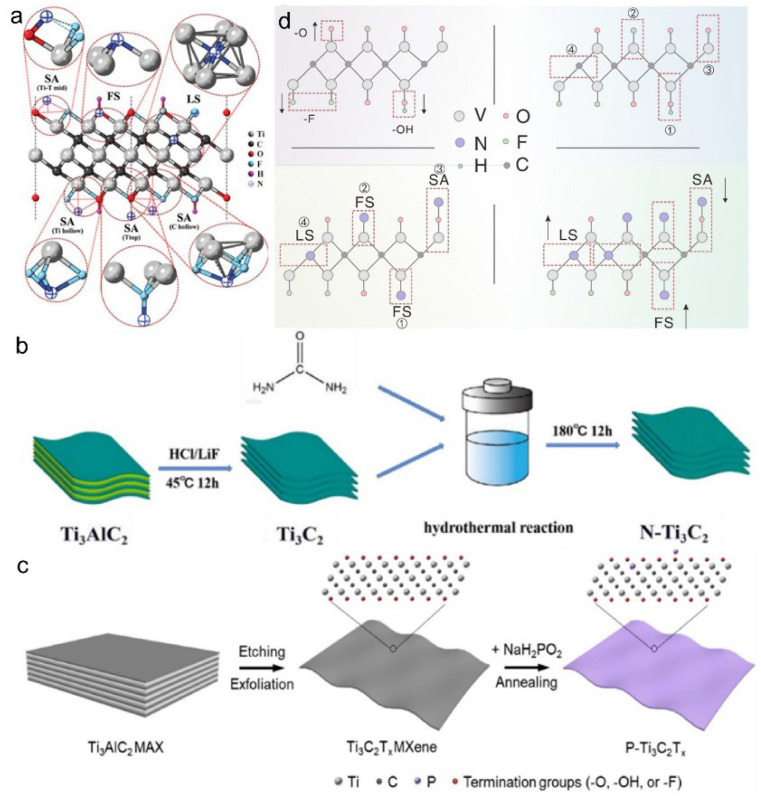
(**a**) Schematic illustration of multiple nitrogen-doping configurations in Ti_3_C_2_T_x_ MXene: surface adsorption (SA), functional group substitution (FS), and lattice substitution pathways (LS). (**b**) Schematic illustration of the hydrothermal nitrogen doping process of Ti_3_C_2_ MXene using urea as the nitrogen source. (**c**) Schematic illustration of the phosphorus doping process of Ti_3_C_2_T_x_ MXene via NaH_2_PO_2_-assisted annealing. Adapted from Ref. [59], with permission from Elsevier. (**d**) Schematic illustration of the surface termination evolution and nitrogen modification process of MXenes. Adapted from Ref. [60], with permission from Elsevier.

Unlike intercalation and surface functionalization, heteroatom doping directly incorporates non-metallic or metallic elements into the MXene lattice. For instance, nitrogen doping increases the electrical conductivity of Ti_3_C_2_T_x_ via surface adsorption (SA), functional group substitution (FS), and lattice substitution (LS) mechanisms (Figure 12a). Each doping mechanism contributes uniquely to the structural stability and electrochemical performance of MXenes [59]. Non-metal doping effectively tailors the physicochemical characteristics of MXenes by inducing lattice distortion, forming ion diffusion pathways, and generating defect sites that serve as electrochemically active centers [9]. Simultaneously, doping can modify surface terminations, such as replacing –F and –OH with –N groups (Figure 12b), thereby enhancing interfacial redox activity and altering surface chemistry.

Moreover, heteroatom incorporation, introduced via thermal, hydrothermal, or UV-assisted treatments. Hydrothermal doping with urea effectively incorporates nitrogen, expands into including nitrogen and phosphorus, and significantly influences the electronic structure of MXenes. For instance, phosphorus doping reduces the work function by 0.8 eV, facilitating improved charge transfer and electrolyte compatibility. Such modifications are commonly the interlayer spacing and improve surface functionalization (Figure 12c). Similarly, annealing Ti_3_C_2_T_x_ with NaH_2_PO_2_ introduces phosphorus atoms while preserving the layered morphology, enabling concurrent modulation of surface terminations and electronic properties (Figure 12d) [60]. Notably, nitrogen doping enriches –OH groups, expands the interlayer spacing to 2.708 nm, and enhances the specific capacitance of the MXene material [61]. To address MXene restacking and the polysulfide shuttle effect in lithium–sulfur batteries, nitrogen doping has also been employed to introduce Ti–N bonds and nitrogen-containing groups that strengthen chemical interactions with Li_2_S_x_ species. This strategy enhances polysulfide immobilization, improves structural stability, and boosts long-term electrochemical performance [62].

**Figure 13:**
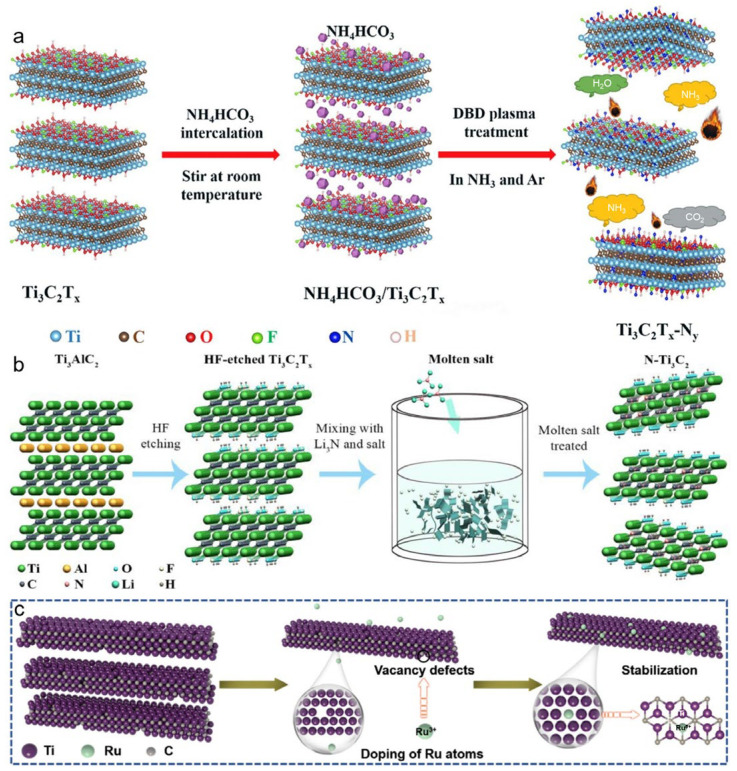
(**a**) Schematic illustration of nitrogen-doped delaminated MXene via NH_3_/Ar plasma treatment, employing NH_3_ gas and NH_4_HCO_3_ as nitrogen sources. Adapted from Ref. [63], with permission from Elsevier. (**b**) Schematic illustration of nitrogen-doped Ti_3_C_2_T_x_ prepared by molten-salt thermal treatment with Li_3_N (lithium nitride) as the nitrogen precursor. Adapted from Ref. [64], with permission from Elsevier. (**c**) Schematic illustration of Ru single-atom anchoring: Ru^3+^ ions electrostatically adsorb onto MXene surface functional groups and are spontaneously reduced by Ti-vacancy defects to form isolated Ru atoms. Adapted from Ref. [18], with permission from American Chemical Society.

For example, nitrogen doping via NH_3_/Ar plasma treatment enables active nitrogen species (such as NH_2_, NH, and N) to react with surface-functional groups, resulting in a nitrogen-doped, layered MXene structure (Figure 13a). This N-doped MXene also exhibits excellent electrochemical performance [63].

An alternative doping route involves molten salt thermal treatment, where MXenes are mixed with nitrogen-rich precursors such as Li_3_N and heated in molten salt environments. During the process, nitrogen atoms are diffused and incorporated into the MXene structure, leading to nitrogen-doped Ti_3_C_2_T_x_ with expanded interlayer spacing and improved ion transport pathways. This method is schematically illustrated in Figure 13b, demonstrating a scalable strategy for achieving bulk nitrogen incorporation under relatively mild reaction conditions [64].

Furthermore, single-atom metal doping strategies have also emerged, exemplified by the anchoring of Ru atoms onto the MXene surface. In this approach, Ru^3+^ ions are electrostatically adsorbed onto functional groups of MXene and spontaneously reduced by Ti vacancy defects, resulting in the formation of atomically dispersed Ru sites. This process not only enhances catalytic activity but also stabilizes the dopants against agglomeration, as shown in Figure 13c [18].

**Figure 14:**
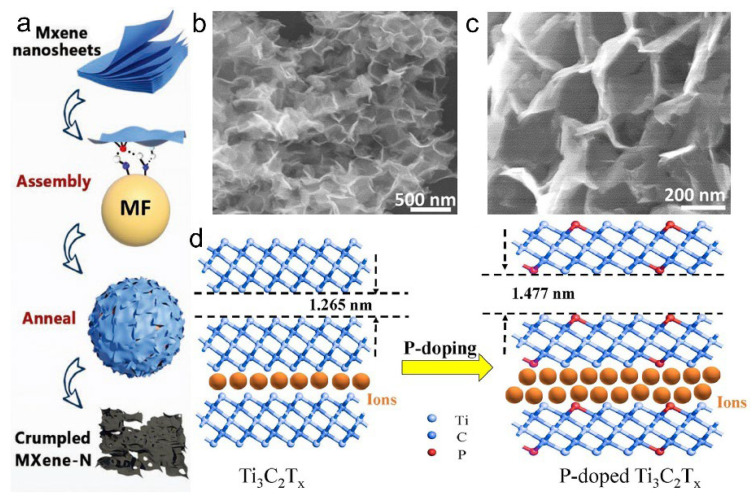
(**a**) Schematic illustration of template-assisted nitrogen doping and morphological engineering of MXene via melamine–formaldehyde (MF) assembly and annealing. (**b**,**c**) SEM image of wrinkled MXene-N nanosheets. Adapted from Ref. [65], with permission from Wiley-VCH. (**d**) Schematic illustration of interlayer expansion and enhanced ion accessibility in Ti_3_C_2_T_x_ MXene induced by phosphorus doping. Adapted from Ref. [66], with permission from Elsevier.

In addition, alternative doping techniques such as template-assisted doping, in which MXene nanosheets are electrostatically assembled with melamine–formaldehyde (MF) templates followed by high-temperature annealing, allow nitrogen atoms to substitute for lattice carbon or bind to surface functional groups (Figure 14a). Upon removal of the MF template, a crumpled and porous MXene structure is formed, effectively preventing sheet restacking while enhancing surface redox activity and conductivity.

The resulting wrinkled morphology of the N-doped MXene is further confirmed by SEM imaging (Figure 14b,c), which reveals a highly folded and interconnected nanosheet architecture favorable for ion transport and electrochemical reactivity [65]. UV-assisted doping has also been employed to eliminate undesirable surface terminations, thereby increasing the capacitive contribution to as high as 86.3%, underscoring its strong potential for high-performance energy storage applications [67].

Beyond nitrogen doping, phosphorus incorporation—due to the larger atomic radius of phosphorus (≈110 pm) compared to nitrogen (≈65 pm)—can introduce lattice distortion and expose additional electrochemically active sites. As a result, phosphorus-doped MXenes have garnered significant research interest. The conventional phosphorus-doping method, which involves mixing Ti_3_C_2_T_x_ with red phosphorus, followed by high-temperature annealing under an argon atmosphere, often leads to the formation of titanium phosphate phases, which are detrimental to electrochemical energy storage.

To overcome this limitation, researchers have developed an in situ crosslinking approach, wherein a Ti_3_C_2_T_x_ colloidal suspension is combined with potassium tripolyphosphate (KTPP). Hydrogen bonding drives the formation of a stable network structure, which prevents restacking. Upon annealing in an inert atmosphere, KTPP decomposes, releasing phosphorus atoms that intercalate into both the surface and interlayer regions, thereby achieving uniform doping without undesirable phosphate phase formation [17]. The effect of phosphorus doping on interlayer spacing and ion accessibility is schematically illustrated in Figure 14d, where P incorporation leads to an increase in d-spacing from 1.265 nm to 1.477 nm [66], facilitating more efficient ion transport. This process optimizes the electronic states of alkali metals within MXenes and reduces the energy barriers for intermediate species, resulting in higher specific capacitance, improved cycling stability, and enhanced energy density [68].

**Figure 15:**
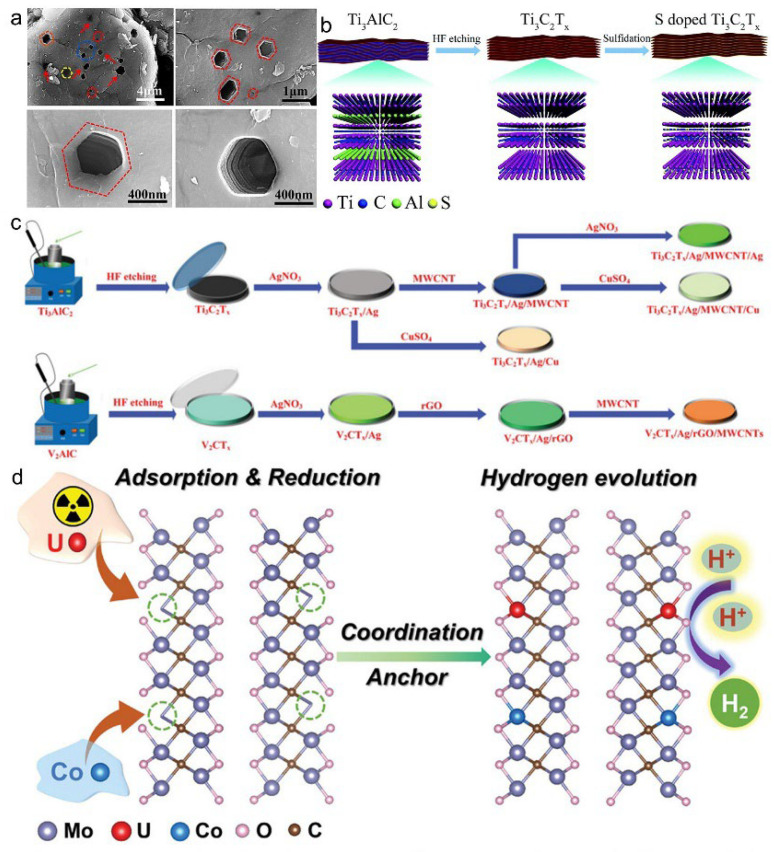
(**a**) Microstructural and morphological features of hexagonal-pore MXene electrode materials regulated by carbon vacancy engineering. Adapted from Ref. [21], with permission from Elsevier. (**b**) Schematic illustration of sulfur doping in Ti_3_C_2_T_x_ MXene via HF etching and sulfuration from Ti_3_AlC_2_ precursor. Adapted from Ref. [69], with permission from Royal Society of Chemistry. (**c**) Schematic synthesis routes of metal- and carbon-based doped MXene composites derived from Ti_3_AlC_2_ and V_2_AlC precursors. Adapted from Ref. [70], with permission from Wiley-VCH. (**d**) Schematic illustration of MXene doped with uranium (U) and cobalt (Co) ions. Adapted from Ref. [71], with permission from Wiley-VCH.

To mitigate the ion transport limitations caused by MXene restacking and structural defects, a defect engineering strategy involving high-temperature synthesis followed by rapid quenching has been developed. This approach transforms disordered carbon vacancies into an ordered network, enhancing structural uniformity. Subsequently, microwave-assisted etching is employed to selectively remove aluminum layers, yielding multilayered MXenes with hexagonal pores, known as V-TC structures (Figure 15a). These architectural modifications shorten ion diffusion paths and increase interlayer spacing, thereby facilitating enhanced ion mobility. Additionally, carbon vacancies reduce charge transfer resistance, further promoting rapid ion migration [21].

Sulfuration serves as an effective modification technique to enhance the electrochemical performance of MXenes, particularly in sodium-ion batteries. By introducing sulfur-containing functionalities onto the MXene surface, Ti–S bonds are formed, which not only enhance electronic conductivity but also expand interlayer spacing, thereby facilitating Na^+^ diffusion. Consequently, sulfur-doped Ti_3_C_2_T_x_ (S–Ti_3_C_2_T_x_) demonstrates significantly improved reversible capacity and cycling stability, delivering 183.2 mAh·g^−1^ after 100 cycles at 0.1 A·g^−1^ and maintaining 76 mAh·g^−1^ after 1500 cycles at 2 A·g^−1^. The transformation process, involving hydrofluoric acid (HF) etching followed by sulfuration, is illustrated in Figure 15b, depicting the conversion of Ti_3_AlC_2_ to S–Ti_3_C_2_T_x_ [69]. Furthermore, low-temperature sulfuration using agents, such as thiourea or Na2S, effectively incorporates sulfur atoms, further enlarging interlayer spacing and promoting ion diffusion. The high electronegativity of sulfur increases the electronic density within the host lattice, thereby improving electrical conductivity and enhancing the overall energy storage performance.

Beyond non-metallic doping and structural defect modulation, incorporating metal ions into MXene-based materials offers additional avenues for property enhancement. A notable example is the development of a hierarchical silver (Ag) and copper (Cu) co-doped MXene/multi-walled carbon nanotube (MWCNT) composite. This composite construct has extensive electronic pathways and optimizes both the interlayer architecture and charge distribution. Specifically, co-doping with Ag and Cu leads to the formation of Ti–Ag bonds and dual-channel Cu–MWCNT networks. These structural modifications increase the conduction channel width from 0.49 nm to 6.58 nm and reduce the bulk resistivity to 9.668 × 10^−7^ Ω·m. The synthetic pathway, illustrated in Figure 15c, details the stepwise formation of metal- and carbon-based doped MXene hybrids from Ti_3_AlC_2_ and V2AlC precursors [70]. In addition to energy storage applications, MXenes have garnered attention in advanced catalytic processes, particularly within the nuclear energy sector. Uranium (U), characterized by its unique 5f orbitals and multiple oxidation states ranging from +1 to +6, has been anchored onto defect-rich MXene surfaces, either individually or in combination with cobalt (Co). These interactions facilitate the tuning of MXene’s electronic structure, thereby enhancing its catalytic activity. Such modifications enable efficient hydrogen evolution and redox processes, contributing to the sustainable transformation of radioactive feedstocks. Figure 15d illustrates this mechanism, showcasing the coordination of U and Co atoms onto molybdenum (Mo)-based MXene frameworks [71].

Recent studies have shown that co-doping with Nb and Ti/V can increase the density of electroactive sites, thereby enhancing electrical conductivity and reducing ion diffusion barriers [72]. In summary, heteroatom doping—whether non-metallic, metallic, or co-doped—precisely tunes MXene’s structure and chemistry, leading to enhanced conductivity, ion transport, and electrochemical performance. These strategies pave the way for advanced MXene-based energy storage and catalytic systems.

### 2.4. Composite Engineering on MXene

Although pristine Ti_3_C_2_T_x_ MXene exhibits high conductivity and abundant surface terminations, it suffers from nanosheet restacking and oxidative degradation, limiting ion accessibility and structural stability. Therefore, composite engineering, through the integration of MXene with carbon-based materials, conductive polymers, or metal oxides, represents an effective strategy to suppress restacking, improve environmental stability, and exploit interfacial synergism for enhanced electrochemical performance. To fabricate various MXene-based composites, a wide range of synthesis techniques has been explored, including physical mixing, ball milling, vacuum-assisted filtration, self-assembly, in situ growth, hydrothermal treatment, electrophoretic deposition, and electrospinning. These methods collectively contribute to enhancing the performance of MXenes in diverse practical applications.

Building upon the strategy of composite engineering, the incorporation of carbon nanotubes (CNTs) into MXene frameworks has garnered significant attention due to the complementary advantages offered by both materials. The integration of MXene with CNTs combines the high pseudocapacitance and catalytic activity of MXenes with the superior conductivity and structural integrity of CNTs, thereby overcoming the intrinsic limitations of each component and expanding their potential in applications such as energy storage, sensors, and electromagnetic interference (EMI) shielding [73,74,75]. A wide range of MXene/CNT composite architectures has been developed—encompassing one-dimensional fibers, two-dimensional films, and three-dimensional aerogels—that effectively optimize both ion diffusion and electron transport pathways [76].

**Figure 16:**
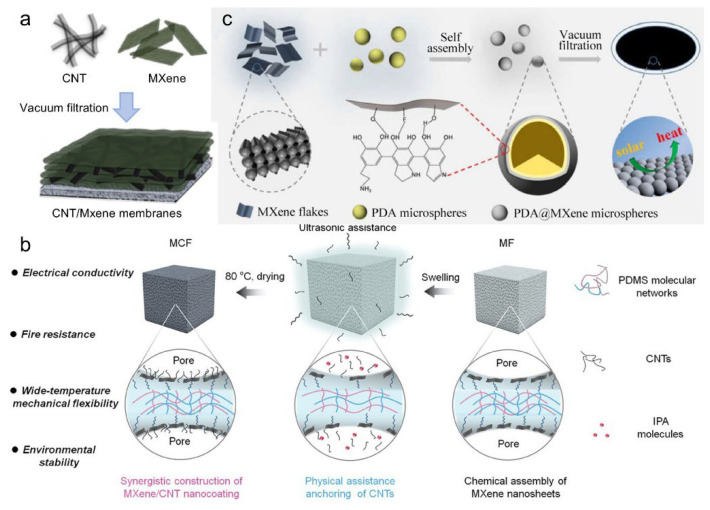
(**a**) Schematic illustration of the fabrication of MXene/CNT composite membranes via vacuum filtration. Adapted from Ref. [78], with permission from Elsevier. (**b**) Schematic illustration of the synergistic assembly of MXene/CNT nanocoatings in porous elastomeric networks. Adapted from Ref. [77], with permission from Wiley-VCH. (**c**) Schematic illustration of the fabrication of PDA@MXene composite microsphere photothermal films via physical mixing, self-assembly, and vacuum filtration methods. Adapted from Ref. [79], with permission from Springer Nature.

Physical mixing is a commonly used strategy for fabricating MXene-based composites, particularly with carbon nanotubes (CNTs), by ultrasonically blending MXene dispersions with CNT suspensions, followed by vacuum filtration [77]. This technique offers simplicity, low cost, and scalability, but often suffers from weak interfacial bonding between MXene and CNTs, leading to limited enhancement in composite performance. As shown in Figure 16a, vacuum filtration enables the formation of layered CNT/MXene membranes, where CNTs intercalate between MXene sheets to reduce restacking and improve conductivity [78].

To overcome interfacial incompatibility in MXene-based composites, a synergistic assembly strategy has been developed, wherein CNTs and MXene nanosheets are co-anchored within porous PDMS elastomer networks via ultrasonic dispersion and drying. This architecture yields nanocoatings with enhanced electrical conductivity, mechanical flexibility, fire resistance, and environmental stability (Figure 16b) [77]. Alternatively, MXene nanosheets can be combined with polydopamine (PDA) through physical mixing, self-assembly, and vacuum filtration to construct PDA@MXene microspheres, which are further processed into photothermal films (Figure 16c) [79]. When embedded in polymer matrices such as PVDF, these structures exhibit efficient solar-to-thermal energy conversion [80]. However, the absence of chemical bonding in physical mixing methods hinders the enhancement of interfacial synergistic effects in MXene-based composites [81,82,83,84].

Among these techniques, mechanical approaches such as ball milling and vacuum-assisted filtration have been widely employed due to their simplicity and scalability. Ball milling is a widely adopted mechanical technique for fabricating MXene–carbon composites [85,86]. This method employs mechanical forces to fracture MXene sheets and ensure homogeneous mixing with carbonaceous materials. Its advantages include high processing efficiency and broad compatibility with diverse carbon-based materials. However, several potential drawbacks must be considered. The localized high temperatures and pressures generated during the milling process may modify the surface functional groups of MXenes, thereby influencing their interfacial interactions with carbon species. Furthermore, excessive milling duration or intensity can induce excessive fragmentation of MXene nanosheets, reducing their specific surface area and potentially degrading the composite’s overall performance.

**Figure 17:**
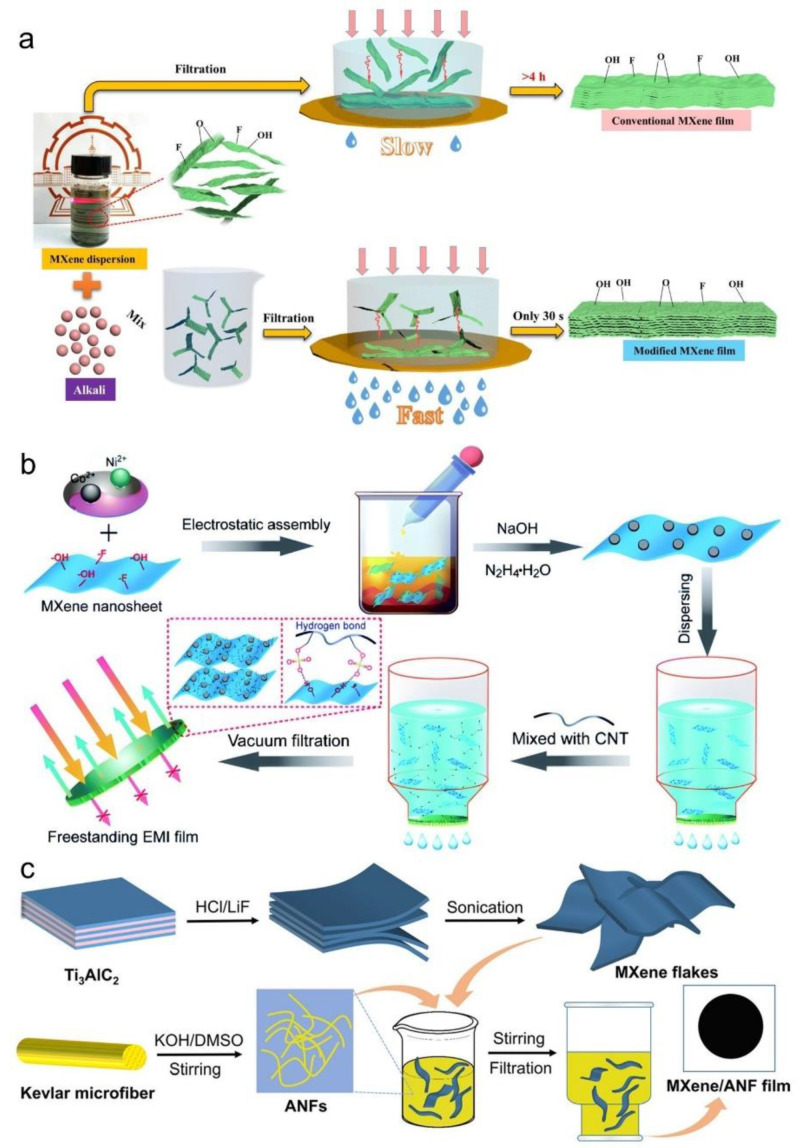
(**a**) MXene film prepared by electrolyte-induced flocculation combined with vacuum-assisted filtration. Adapted from Ref. [88], with permission from Multidisciplinary Digital Publishing Institute. (**b**) Schematic illustration of the synthesis process for MXene/CNT-based freestanding films via electrostatic assembly and vacuum-assisted filtration for EMI shielding applications. Adapted from Ref. [91], with permission from Royal Society of Chemistry. (**c**) Schematic illustration of the preparation process of MXene/ANF composite films. Adapted from Ref. [92], with permission from American Chemical Society.

Vacuum-assisted filtration is another effective method that utilizes negative pressure to facilitate the layer-by-layer assembly of MXene onto composite substrates, resulting in films with well-defined and controllable architectures [87]. One notable advancement in this area is the electrolyte-induced flocculation strategy, which significantly accelerates the filtration process by introducing alkali ions to neutralize surface charges on MXene nanosheets. As shown in Figure 17a, this approach enables the rapid formation of MXene films—within just 30 s—compared to over 4 h for conventional filtration, while preserving structural order and minimizing surface functional group degradation [88]. This technique is particularly advantageous for fabricating flexible films with tailored microstructures. For instance, MXene/aramid nanofiber (ANF) composite films prepared via this approach demonstrated superior flexibility and outstanding electromagnetic interference (EMI) shielding capabilities [89,90].

In addition to enhanced filtration kinetics, the combination of electrostatic self-assembly and vacuum-assisted filtration has also shown promise in improving film uniformity and interfacial compatibility. For instance, as illustrated in Figure 17b, modified MXene nanosheets are electrostatically assembled with Ni^2+^ ions and dispersed carbon nanotubes (CNTs), followed by vacuum filtration to produce freestanding EMI shielding films. This approach leverages the combined effects of hydrogen bonding and charge-driven interactions to construct material frameworks with outstanding electrical conductivity and electromagnetic attenuation efficiency [91].

Moreover, vacuum-assisted filtration has been successfully applied in constructing MXene/polymer composite films, such as those based on aramid nanofibers (ANFs). As depicted in Figure 17c, Ti_3_C_2_T_x_ MXene flakes obtained via selective etching and sonication are blended with Kevlar-derived ANFs—prepared through KOH/DMSO treatment—followed by stirring and filtration. The resulting MXene/ANF films exhibit excellent flexibility, mechanical reinforcement, and high EMI-shielding effectiveness, demonstrating the versatility of this approach for fabricating multifunctional MXene-based materials [92].

**Figure 18:**
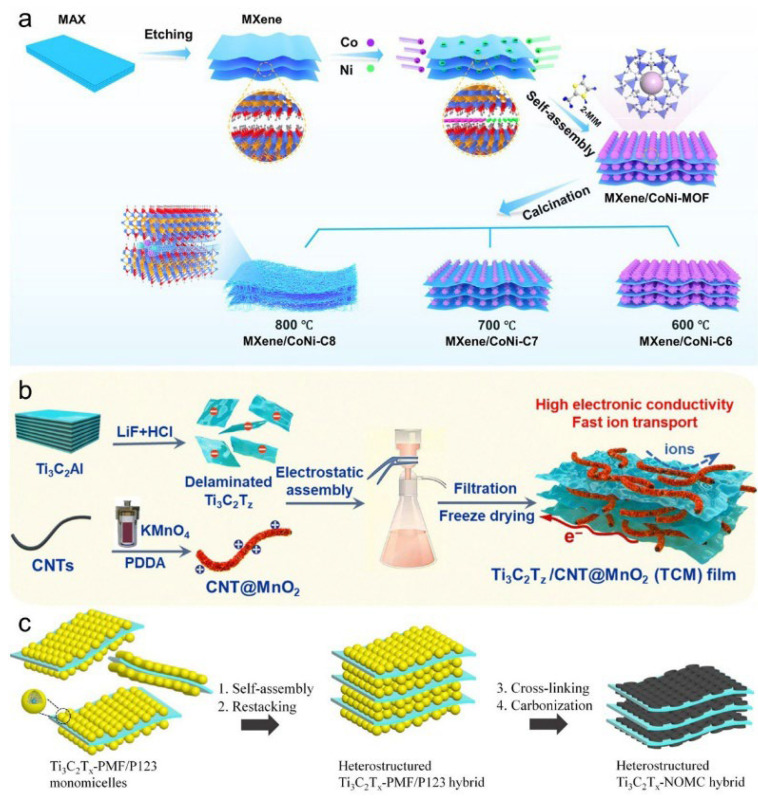
(**a**) Schematic illustration of the process for preparing three-dimensional MXene/CoNi-MOF heterogeneous composites via the self-assembly method. Adapted from Ref. [82], with permission from Elsevier. (**b**) Schematic illustration of electrostatic self-assembly and freeze-drying fabrication of Ti_3_C_2_T_x_/CNT@MnO_2_ (TCM) composite film. Adapted from Ref. [98], with permission from Royal Society of Chemistry. (**c**) Schematic synthesis of heterostructured Ti3C2Tx–NOMC hybrid via Ti3C2Tx–PMF/P123 monomicelle self-assembly, restacking, cross-linking, and carbonization. Adapted from Ref. [93], with permission from Elsevier.

Although mechanical methods are effective for fabricating MXene composites, their limited control over the microstructure and interfaces has prompted growing interest in self-assembly strategies for enhanced structural tunability. The self-assembly method has been effectively utilized to construct three-dimensional MXene/CoNi-MOF heterogeneous composites, as illustrated in Figure 18a. In this strategy, delaminated MXene nanosheets were mixed with cobalt and nickel precursors to assemble a CoNi-based metal–organic framework (CoNi-MOF), followed by calcination at various temperatures (600–800 °C). The resulting MXene/CoNi-C hybrids exhibited adjustable microstructures and phase compositions, allowing for the optimization of their electrochemical performance. This method relies on electrostatic interactions, such as the binding between positively charged CNT-PEI and negatively charged MXene nanosheets, to form stable and well-organized hybrid architectures [93,94,95,96,97].

This strategy is straightforward and offers controllable interlayer spacing, which enhances ion diffusion pathways and structural stability. As depicted in Figure 18b, Ti3C2Tx nanosheets were electrostatically assembled with δ-MnO_2_-coated carbon nanotubes (CNT@MnO_2_), followed by filtration and freeze-drying. The resulting freestanding Ti_3_C_2_T_x_/CNT@MnO_2_ (TCM) film demonstrated excellent electronic conductivity, efficient ion transport, and superior electrochemical performance, including high specific capacitance and rate capability [98]. Nevertheless, this method requires precise control over solution pH and surface functional groups, which poses challenges for large-scale application.

Extending this approach, a recent study utilized phenolic resin (PMF) and the triblock copolymer P123 as soft templates to fabricate Ti_3_C_2_T_x_–PMF/P123 monomicelles through spontaneous assembly and restacking (Figure 18c). Subsequent hydrothermal crosslinking and high-temperature carbonization yielded a heterostructured Ti_3_C_2_T_x_–NOMC hybrid featuring nitrogen-doped active sites and ordered mesoporous channels, significantly enhancing charge storage capacity and catalytic performance [93].

**Figure 19:**
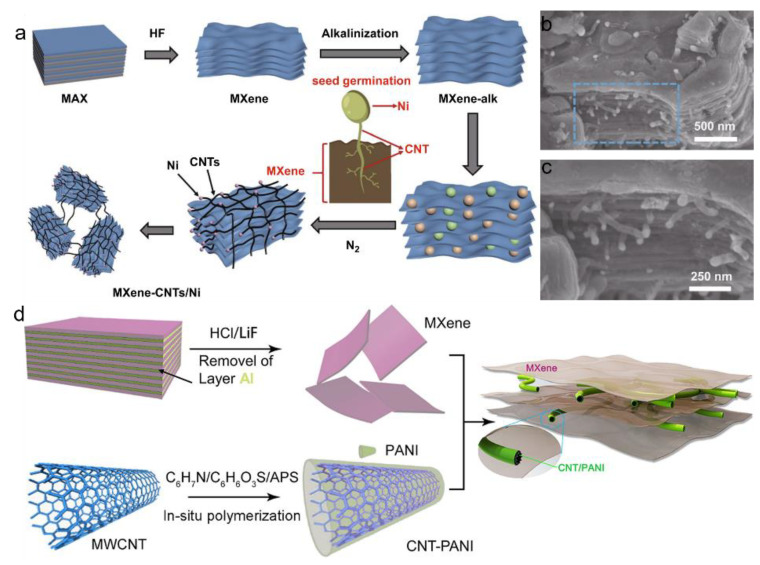
(**a**) Schematic diagram of the in situ growth process for 3D MXene-CNTs/Ni composite. (**b**,**c**) SEM images of MXene-CNTs/Ni composite. Adapted from Ref. [102], with permission from Springer. (**d**) Schematic diagram of the in situ growth process for MXene-CNT/PANI composite. Adapted from Ref. [81], with permission from Elsevier.

The in situ growth method involves the catalytic generation of carbon nanostructures directly on MXene surfaces, typically by preloading metal catalysts such as Fe or Co, followed by chemical vapor deposition (CVD) to grow carbon nanotubes (CNTs) [99,100,101]. This approach enables uniform composite distribution and robust interfacial bonding, leading to significantly enhanced electrical conductivity and mechanical strength. As shown in Figure 19a, Ni nanoparticles are seeded on alkalized MXene surfaces, initiating CNT growth directly from the MXene substrate. This strategy produces an interconnected MXene–CNTs/Ni hybrid structure through a root-like growth mechanism, where CNTs bridge adjacent MXene layers, improving charge transport pathways and mechanical integration. However, it is characterized by complex processes and high-temperature requirements. The corresponding SEM images further confirm the effectiveness of this structure. As illustrated in Figure 19b,c, the in situ-grown CNTs are uniformly distributed across the MXene surface, forming a three-dimensional conductive network that enhances the accessible surface area and supports electrolyte infiltration [102].

Beyond CNTs, the in situ growth method can also be extended to hybrid systems incorporating conductive polymers. For instance, Figure 19d shows a schematic of the synthesis of MXene/CNT–PANI composites, where polyaniline (PANI) is polymerized onto CNTs via in situ chemical oxidation. These CNT/PANI hybrids are subsequently integrated with MXene nanosheets, forming a multilayered architecture with interpenetrating conductive networks and enhanced structural stability [81]. This configuration effectively combines the high pseudocapacitance of PANI, the mechanical flexibility of CNTs, and the surface-rich MXene structure, yielding multifunctional composites suitable for high-performance energy storage applications.

**Figure 20:**
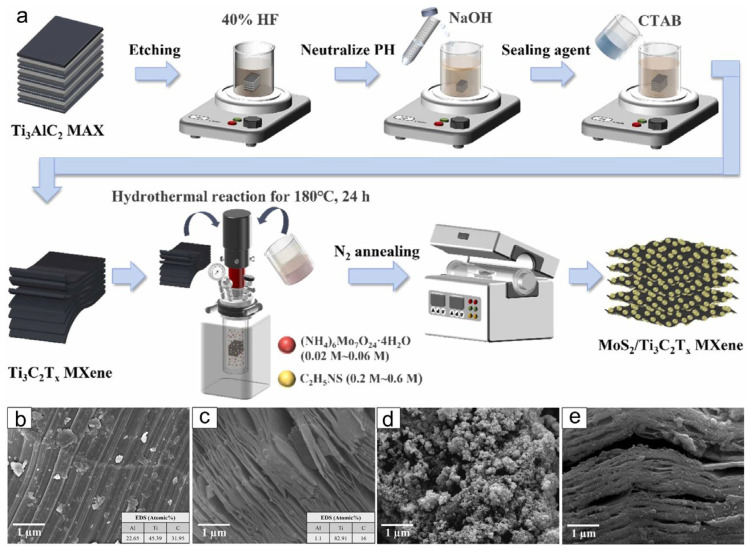
(**a**) Schematic illustration of the MoS_2_/MXene electrode synthesized via the hydrothermal method. FE-SEM images of (**b**) MAX; (**c**) MXene; (**d**) MoS_2_ electrodes and (**e**) MoS_2_/MXene electrodes. Adapted from Ref. [106], with permission from Elsevier.

In addition to in situ catalytic growth strategies, solution-based techniques such as the hydrothermal method have also been widely employed to fabricate MXene-based composites with three-dimensional architectures. The hydrothermal method utilizes a solvent-based thermal reaction to promote the in situ growth of carbon nanotubes (CNTs) or other functional nanomaterials on MXene, forming a three-dimensional (3D) composite structure [103,104,105]. As illustrated in Figure 20a, a typical hydrothermal synthesis process involves the etching of Ti_3_AlC_2_ to obtain a delaminated Ti_3_C_2_T_x_ MXene, followed by mixing with molybdenum and sulfur sources in an autoclave at 180 °C for 24 h, and subsequent N_2_ annealing to produce MoS_2_/Ti_3_C_2_T_x_ composites. The morphology and structural evolution of the materials throughout this process are confirmed by field-emission scanning electron microscopy (FE-SEM).

The pristine MAX phase (Ti_3_AlC_2_) shows a layered lamellar morphology with compact stacking (Figure 20b), which transitions to a more exfoliated sheet-like structure after HF etching to produce MXene (Figure 20c). The MoS_2_ nanosheets formed during hydrothermal synthesis exhibit a flower-like morphology with abundant surface roughness (Figure 20d), while the final MoS_2_/MXene composite displays a loosely packed, layered architecture, indicating successful integration of MoS_2_ into the MXene matrix (Figure 20e) [106]. These MoS_2_/MXene composites have been applied in high-performance supercapacitors, exhibiting high specific capacitance and excellent cycling stability. Furthermore, by tuning the sulfur source and post-hydrothermal annealing parameters, MXene–MoS_2_ composites have also been developed for efficient pesticide removal applications, showcasing their multifunctional environmental adaptability [107].

**Figure 21:**
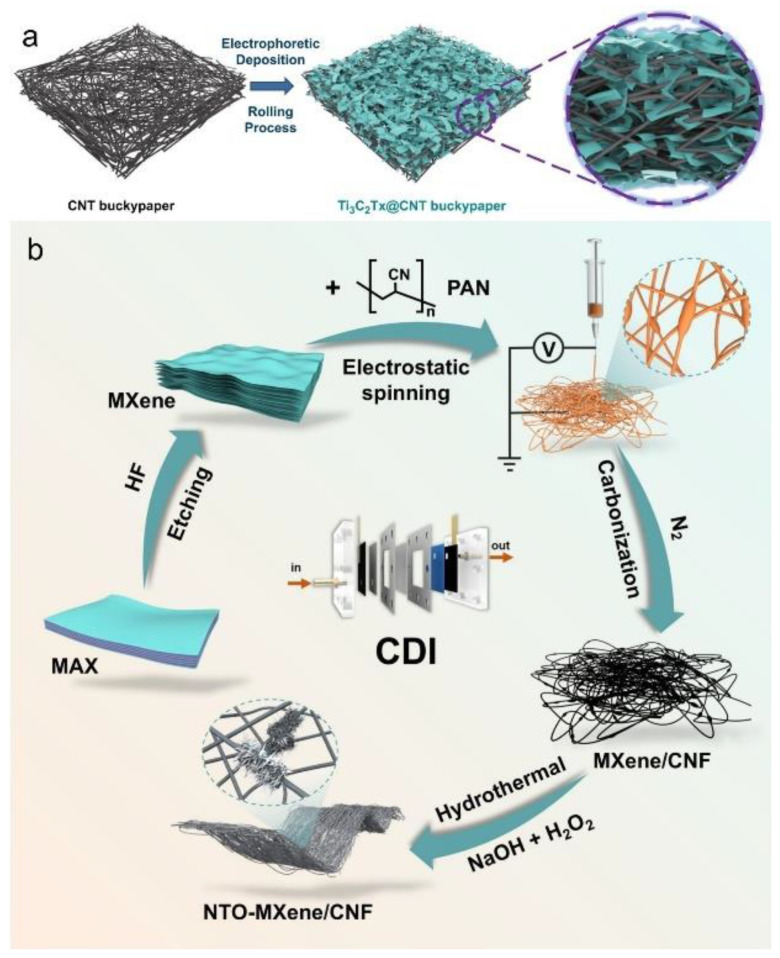
(**a**) Schematic illustration of the preparation of the Ti_3_C_2_T_x_@CNT composite via the electrophoretic deposition. Adapted from Ref. [110], with permission from Springer. (**b**) Schematic illustration of the synthesis of a binder-free NTO (sodium titanate)-MXene/CNF film. Adapted from Ref. [113], with permission from Elsevier.

Electrophoretic deposition (EPD) is a promising technique wherein charged MXene (Ti_3_C_2_T_x_) nanosheets are driven by an external electric field to migrate and form uniform, binder-free coatings on conductive carbon nanotube (CNT) frameworks. This process yields mechanically robust composite electrodes with enhanced interfacial contact and improved electrochemical performance [108,109,110]. As shown in Figure 21a, CNT buckypaper serves as a conductive scaffold onto which Ti_3_C_2_T_x_ nanosheets are electrophoretically deposited, followed by a rolling process to ensure intimate contact and densification. The resulting Ti_3_C_2_T_x_@CNT composite demonstrates a well-organized architecture with embedded MXene flakes, effectively overcoming the aggregation and restacking issues commonly observed in traditional fabrication routes [111]. This method enables rapid, large-area film formation without the use of binders or charge-inducing agents and significantly enhances electrode reliability. Moreover, uniform MXene deposition onto carbon fiber surfaces via EPD has been reported to improve both interlaminar shear strength and flexural properties, while avoiding the damage and complexity of conventional coating techniques [112]. However, the limitations of this approach include the requirement for specialized equipment and its restriction to conductive substrates.

Electrospinning, another scalable fabrication method, involves the electrostatic spinning of a homogeneous polymer/MXene dispersion into nanofibers, enabling the formation of flexible composite membranes [113,114,115]. This technique offers key advantages, such as a high surface-area-to-volume ratio and controllable fiber morphology. As illustrated in Figure 21b, MXene nanosheets are first exfoliated from MAX precursors via HF etching and then integrated with polyacrylonitrile (PAN) through electrospinning. After carbonization under a nitrogen atmosphere, the resultant MXene/carbon nanofiber (CNF) network undergoes hydrothermal treatment in a NaOH and H_2_O_2_ solution to yield a sodium titanate (NTO)-MXene/CNF composite film. This hierarchical architecture provides interconnected conductive pathways and porous structures that are ideal for capacitive deionization (CDI) systems. Additionally, the uniform dispersion of MXene in polymer matrices significantly enhances mechanical strength and thermal conductivity [116].

MXene-based composites have demonstrated significant potential in electrochemical energy storage and conversion beyond conventional supercapacitor applications. Metal–organic frameworks (MOFs), known for their high surface area and tunable porosity, are often limited by poor electrical conductivity and structural fragility. Conversely, MXenes possess excellent conductivity but tend to restack, hindering ion transport. To overcome these challenges, in situ growth strategies, such as encapsulating MXenes with MOFs, have been developed to form three-dimensional heterostructures like Ni-MOF@MXene, which exhibit enhanced capacitance and improved structural stability [117]. Similarly, Ti_3_C_2_/Si composites effectively accommodate the volumetric changes of anodes during cycling, thereby enhancing both cycling stability and rate capability [118]. The combination of MXenes with carbon-based materials, such as carbon nanotubes (CNTs) and reduced graphene oxide (rGO), through electrostatic self-assembly or vacuum-assisted filtration, facilitates the construction of three-dimensional conductive networks that suppress MXene restacking and promote efficient electron and ion transport. In addition, the in situ hydrothermal growth of CoSe_2_ nanorods on MXene produces hierarchical conductive networks, wherein CoSe_2_ provides both chemical adsorption sites and catalytic centers via the formation of S–Co and Li–Se bonds. The synergy between the catalytic activity of CoSe_2_ and the intrinsic conductivity of MXene significantly improves lithium polysulfide conversion, offering a promising strategy for next-generation lithium–sulfur batteries [119].

**Figure 22:**
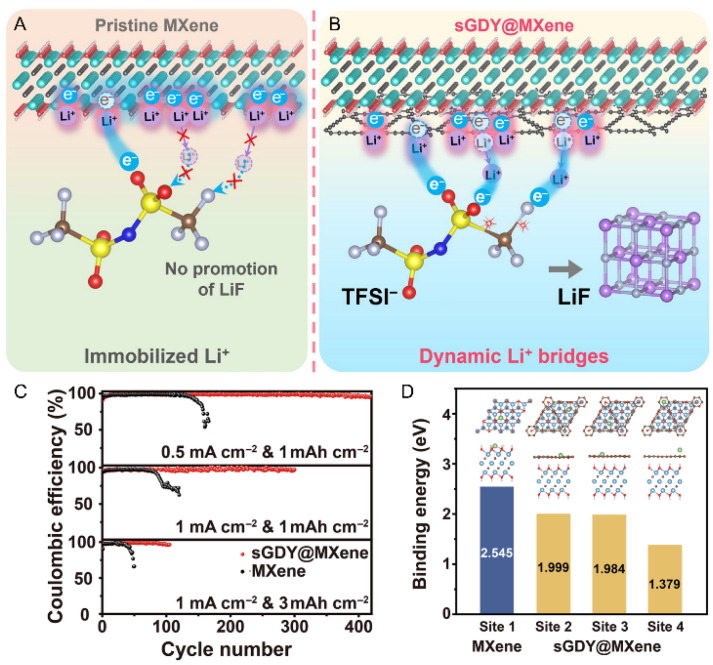
(**A**,**B**) Schematic illustration of the charge transfer process mediated by lithium-ion bridging, facilitated by sGDY@MXene, which regulates the formation of dynamic Li^+^ bridges through LiF. (**C**) Comparison of the Coulombic efficiencies of pristine MXene and sGDY@MXene under different current densities and areal capacities. (**D**) Calculated Li^+^ binding energies at different stable adsorption sites. Adapted from Ref. [120], with permission from Springer.

To further enhance interfacial ion transport and address the limitations of traditional MXene architectures, recent efforts have focused on molecular-level engineering strategies. Notably, functionalizing MXene with sulfur-doped graphdiyne (sGDY) has led to significant improvements. As depicted in Figure 22A,B, pristine MXene exhibits limited promotion of LiF formation, resulting in lithium-ion immobilization at the electrode–electrolyte interface. In contrast, the sGDY@MXene composite facilitates the formation of dynamic Li^+^ bridges, thereby regulating interfacial charge transfer and promoting uniform LiF deposition. This enhancement is evidenced by a Coulombic efficiency of 99.2% at a current density of 1 mA·cm^−2^ and an areal capacity of 1 mAh·cm^−2^, outperforming pristine MXene, especially under high-capacity and high-rate conditions (Figure 22C) [120]. Furthermore, first-principles calculations indicate that Li^+^ binding energies at sGDY@MXene adsorption sites are significantly lower than those on pristine MXene, suggesting more favorable and stable Li-ion accommodation (Figure 22D).

Building upon these strategies, researchers have also explored MXene-based composites in potassium-ion battery applications. For example, embedding Re_2_Te_5_ nanoparticles into MXene constructs a three-dimensional amorphous/crystalline heterointerface, resulting in excellent structural and electrochemical stability. This design enables the electrode to deliver a capacity of 162.5 mAh·g^−1^ at 20 A·g^−1^ with a minimal capacity decay rate of only 0.004% over 5000 cycles [121]. To further enhance electrical conductivity and prevent restacking, MXene has been composited with conductive carbon-based materials, such as carbon spheres (CSs@Ti_3_C_2_) and nitrogen-doped CNTs (MXene@CNTs), which create fast electron transport channels and expose more active sites, thereby improving the electrochemical performance of ion batteries.

Beyond potassium-ion batteries, MXene-based composites have exhibited broad adaptability across various battery chemistries. Hybridizing MXene with manganese compounds (e.g., MnO_2_@V_2_CT_x_, ZnMn_2_O_4_@Ti_3_C_2_T_x_), vanadium oxides (e.g., VO_2_@MXene, V_2_O_5_@Ti_3_C_2_T_x_), and organic materials (e.g., PANI@Ti_3_C_2_T_x_) significantly enhances energy storage performance [122]. In Mn-based systems, MXene buffers volume changes, improves electron transport, and stabilizes active components. In vanadium-based hybrids, its layered structure and surface terminations facilitate Zn^2+^ diffusion while mitigating vanadium dissolution. Organic/MXene composites promote H^+^/Zn^2+^ co-intercalation, boosting zinc-ion battery capacity. Additionally, a VS_2_/Ti_3_C_2_T_x_ heterostructure—combining the redox activity of VS_2_ with MXene’s flexibility—forms a freestanding film that expands interlayer spacing, inhibits VS_2_ stacking, and enhances Zn^2+^ transport, achieving improved reaction kinetics and capacity [123].

Overall, composite engineering effectively addresses the limitations of pristine MXenes by enhancing interfacial interactions and structural stability within the composites, thereby improving their electrochemical performance. The continued development of this strategy will facilitate the advancement of high-performance energy storage systems.

## 3. Conclusions and Outlook

In summary, MXene materials exhibit remarkable potential for energy storage applications, owing to their unique two-dimensional structure and excellent physicochemical properties. Various modification strategies, including intercalation, surface functional group regulation, doping, and composite material incorporation, significantly enhance their electrochemical performance. The pros and cons of each strategy and their possible applications are displayed in Table 1.

This review highlights the pivotal role of structural modulation and surface modification in optimizing MXene materials for energy storage applications. The insights gained here are not only significant for improving the performance of MXenes but also for the broader field of advanced materials for energy conversion and storage, where similar strategies could be applied to other two-dimensional materials. As the demand for high-performance energy storage systems continues to grow, the continued exploration of MXene modifications will be essential in developing next-generation batteries, supercapacitors, and other energy devices with superior efficiency and stability.

Despite significant advances in MXene modification, challenges remain in precisely controlling surface functional groups, ensuring long-term stability under realistic conditions, and developing scalable and cost-effective synthesis methods for commercial applications. Future research should focus on advanced nanofabrication techniques, such as atomic layer deposition, to precisely tune surface chemistry and enhance oxidation resistance. Combining MXenes with other 2D materials, conductive polymers, and metal oxides in hybrid architectures will further improve the energy storage capacity, rate performance, and durability. Moreover, integrating experimental studies with computational modeling can accelerate the rational design of MXene-based materials. Cross-disciplinary collaboration in materials science, electrochemistry, data science, and engineering will open new applications for MXenes in flexible electronics, environmental remediation, and catalysis, helping unlock their full potential in next-generation energy storage systems.

## Figures and Tables

**Table 1:** Summary of MXene modification strategies.

Modification Strategy	Key Advantages	Key Drawbacks	Typical Applications
Intercalation	Enlarges interlayer spacing to mitigate restacking Accelerates ion diffusion kinetics	Potential introduction of impurities Limited control over uniformity	Li^+^/Na^+^/K^+^ batteries; high-rate supercapacitors; ion sieving
Surface Functionalization	Tailors surface chemistry to enhance electrical conductivity and hydrophilicity Improves environmental stability and cycling life	Functional-group instability under harsh environmental conditions Complex processing requirements	High-power electrodes; Li–S battery separators; electrocatalysis
Doping Engineering	Modulates electronic structure to increase carrier density Creates defect-rich active sites	Stringent synthesis conditions (e.g., high-temperature, plasma) Challenges in achieving homogeneous doping	Metal-ion batteries; advanced catalysts; low-temperature storage
Composite Engineering	Synergistic interaction with polymers, CNTs, and oxides enhances mechanical and electrical integrity Suppresses oxidation and restacking	Complex interface control Higher cost and scalability challenges	Flexible/stable electrodes; EMI shielding; photothermal films

## Data Availability

No new data were created or analyzed in this study. Data sharing is not applicable to this article.
